# Report on a Memoir

**Published:** 1817-01

**Authors:** Baron Larrey


*For the London Medical and Physical Journal.*

*Report on a Memoir, by* Baron Larrey—"*Notice on the Effect of Balls lost in the Cavity of the Thorax.*" Read at the Royal Institute on the 21st of October, 1816.

BARON LARREY has been employed in military surgery during the last twenty-five years. He has made every campaign, from the burning sands of Egypt to the frozen deserts of Russia, always as surgeon-in-chief of armies the most numerous, the most victorious, and those which

 have have experienced the most cruel vicissitudes that modern history can recount. No surgeon has ever had, or perhaps ever will have, so many and such vast opportunities of exercising the most active and the most important branches of surgery; and assuredly none will ever exhibit more zeal, skill, or humanity, than Baron Larrey.

He has already collected, in 3 volumes octavo,* (which he has presented to the Academy,) a number of facts, which demonstrate great improvements in the art.

The labours which he has to-day presented, under the modest title of a *Notice*, is not less interesting. It may be regarded as the further development of what he has printed in his collection on the effects of the empyèma: it has, nevertheless, a special object.

The author had already developed, with the sagacity of learning and experience, the effects of an extravasated fluid, when lodged in the cavity of the thorax, during a longer or shorter period; or when a salutary operation gives, or has given, an issue to this fluid.

I will not repeat here what may be read in the work itself, only remarking that the Baron Larrey had already observed the resources which nature employs to fill the immense void left in the breast by the evacuation of the pus or other fluid which it contained,—a void occasioned as well by the contraction of the lung upon itself as by the retrocession of the hard and soft parts which surround the breast, and of the viscera it contains, or are contiguous to it.

He had given the most salutary advice, according to the best precepts of the art, in order to diminish the ill effects of the entrance of the air, whether depending on the compression of the lungs, or the inflammation, suppuration, or the decay of the membranes which line the capacity and envelope the lung.

Though wounds penetrating the breast, and the extravasated matter which is the consequence, are always dangerous, yet it must be admitted that the operation for the empyèma

---

* Memoires de Chirurgie Militaire et Campagnes, de D. J. Larrey, First Surgeon of the Emperor's Guard, Baron of the Empire, Commander of the Legion of Honour, &c. &c.; Inspector General of the Service of Health of the Armies, &c. &c. 3 vols. plates. This invaluable work, now in the course of translation, will comprise the author's MS. notes on the campaign in Russia, which present circumstances will not permit to be published in France. They contain all the remarkable phenomena presented by that unparalleled campaign. The translation may be expected to appear in the course of the winter.

which which relieves the principal disorder, becomes a new one, so dangerous that, notwithstanding all the care of Baron Larrey, he has lost the greater number of his patients.

It nearly always happens that particular and foreign causes intervene, to which are to be attributed the fatal termination. One falls from the repercussion of the suppuration, and the cutaneous perspiration produced by the impression of a cold and humid air. A second owes a relapse to intemperance. A third survives a year, and falls into a mirasmus. A fourth, less favoured, dies in a few days, from the effects of the putrefaction of the extravasated blood, which the operation has not perfectly extracted; or from an internal hæmorrhage, of which it is impossible to discover the source: but it is very evident that all these phenomena (epiphænomines) relate to the primitive disorder; that they happen during the long space of time which even the most fortunate treatment requires, and which would infallibly lead to a cure. In fact, it requires a long time to produce that modification of the membranous coats of the thorax which attracts them to the centre, and directs them toward the internal organs,—while these latter, becoming capable of developing themselves, extend likewise towards the membranous lining of the cavity; and thus, together, concur to fill up the void left by the extraction of the vast quantity of extravasated matter whose presence had deranged the natural organization of the parts, and, by its pressure, had contracted the lung in a manner irreparable, except by the long exertion we have already noticed.

It is this exertion on the part of nature, the continuation of which can alone lead to a cure, that Baron Larrey has discovered and observed, which he has accurately described in the memoir now under consideration, and which offers, at the same time, an example of the resources of art, when a foreign body retained in the thorax produces a surface of suppuration.

A young non-commissioned officer received, in a combat under the walls of Paris, a ball which carried away the upper edge of the fourth rib, on the right side, crossed the lung, and stopped, probably, in the vicinity of the vertebræ. The wound was extremely dangerous, attended with hæmorrhage by the mouth and the wound, and the pain was so extreme that the patient was in great danger of expiring. He was conveyed to the Hospital of Gros Ceillou, three months after receiving the wound, in August, 1814. He had, on the upper part of the right side of the breast, a fistulous wound, connected with the formation of matter in the pectoral cavity, from which exuded pus, on laying on the the right side, with his head hung down over the side of the bed. This suppuration, and the slow fever which accompanied it, had reduced the young man to the last state of marasmus. Baron Larrey probed the wound, and discovered that it descended to the eighth or ninth rib, where he touched a hard metallic body, which could be no other than the ball. He decided on making an opening between the eighth and ninth ribs, which were much dilated, instead of the operation of the empyèma. There exuded three basons (palettes) of pus, which permitted the extraction of the ball. Some accidents took place, but were soon calmed. The third day the pus ceased to issue by the fistulous wound, which was soon healed. Sleep and appetite returned; the quantity of pus issuing from the new wound diminished daily; the side of the breast affected gradually sunk in, and the nipple descended two fingers' breadth; the intercostal spaces diminished in proportion, and a probe entered the wound with difficulty; and the patient seemed to be cured, when he drank brandy to excess, which produced acute pains and a burning fever, which carried him off three months after the operation, and six after receiving the wound.—On opening the body, the upper wound was found cicatrized, and the seat of the disease confined to a very small distance from the lower wound. The pleura costalis had acquired an extraordinary thickness. The mediastinum was directed towards the wounded side. A spongy mass, formed, doubtless, from the cellular membrane of the lungs obliterated, filled the upper part of the thoracic cavity. The two ribs between which the operation had been performed approached each other, towards which the rest of that side tended, and were becoming round instead of flat.

We have seen this piece, which was presented to the Academy; and, if it shews us the attempts of Nature in operating a cure, it shews us, at the same time, how difficult it is to attain it.

The thing is not, however, impossible to the skill and perseverance of Baron Larrey, who knows so well how to multiply and to modify the resources of his art, as occasion demands, of which the following case affords a signal proof.

Louis Claye, æt. 26, received a gun-shot wound at the battle of Mohilow, in Russia, the 22d July, 1812. The ball entered the breast by the interval of the eighth and ninth ribs of the right side, and, coming from a great distance, it lodged in this part of the pectoral cavity. The wounded man lay two days on the field of battle before he was carried to the Hospital. He had lost a great quantity of blood, the flow of which was at first stopped by weakness, and afterwards wards by the pieces of his uniform which the ball had carried in with it, and which the blood had driven back to cover the wound; but he continued to spit blood, and he was nearly suffocated by the mass of this fluid in the breast; when a surgeon, enlarging the wound, extracted several pieces of the uniform, and gave vent to about a quart of sero-sanguine matter, mixed with several black clots of blood. The patient was restored to life by this operation, which was succeeded by only simple dressings; and, in a few months after, he was in a condition to be transported successively to the hospitals of Cowno, Köningsberg, and Thorn. The wound closed spontaneously; but new symptoms of oppression of the chest appeared, and yielded to the spontaneous opening of an abscess formed on the edge of the false ribs, and which gave issue to several pieces of cloth. This abscess cleansed itself, and was cured; but the wound in the intercostal space re-opened, which was dressed in the usual manner, and a fillet of linen kept upon it, in the persuasion that the ball still remained in the seat of the disorder. Yet this man, after being conveyed from hospital to hospital, passed two years at home, and four years after his wound he was received at the hospital of Gras Caillou, viz. on the 19th June, 1816.

Baron Larrey probed the wound, and discovered the ball in the bottom of the right cavity of the breast. The orifice was small, the suppuration abundant, and the patient had become hectic. Yet the Baron determined on extracting the ball; but the intercostal space was so diminished that the ribs nearly touched each other; so that there was no other resource than the section of one of the ribs. This operation, which was very difficult, and also dangerous on account of the proximity of the intercostal artery, suggested the idea of cutting away the upper part of the lower rib; which was obliged to be done to such a depth that the rib was nearly cut through (and on the lower edge of it runs the intercostal artery), in order to introduce the forceps to extract the ball, which was at length obtained, but not without much difficulty. The patient supported the operation with great courage. The wound was dressed, and bandaged in the ordinary manner; and the administration of antispasmodics calmed the irritation.

The seventh day after the operation, (all going on well,) the patient, stooping to ease nature, broke the fragment of the rib operated upon, and the rupture of the intercostal artery produced a dreadful hæmorrhage, which was at length stopped; but the patient had no pulse, his members were cold as death, and death itself seemed approaching. Cordials,

 good good wine, and frictions of æther, restored him a little. Symptoms of low fever followed: his strength was exhausted; he felt dreadful pains in the region of the liver; and the wound, which assumed a very bad appearance, became very painful. Large blisters were applied around it; an emetic produced copious evacuations; appropriate dressings, and the use of bark, and other tonics, at length removed the alarming symptoms. His strength gradually increased; the wound cleansed itself; the suppuration was abundant, and of a good quality; and, at the end of thirty days, the patient was discharged cured.

We have seen this man in a good state of health, in spite of his being extremely wasted by the recent disorder; and we have ascertained the following picture, drawn by the Baron, to be strictly correct.

"The void resulting from the principle of evacuation of the extravasated fluids in this cavity, with the exception of the passage from the seat of the fistulous wound, was gradually filled by the process of contraction, to which the coats of the breast had been subjected, during the four years which had passed from receiving the wound. In fact, the ribs have lost their curvature, as in the first case; and are, doubtless, become cylindrical. The sternum is more flattened on this side than on the left. The diaphragm and the liver are considerably elevated in the thoracic cavity: this elevation has rapidly increased, as well as the approximation of the other parts, since the operation, no longer experiencing any obstacle. These phenomena are characterised by the sinking-in of that side of the breast, the situation of the right nipple, and the void space perceivable under the edge of the false ribs, where, before the operation, the part was distended by the liver, &c."

This picture, traced by the Baron, shows, not only the wonderful resources of his art, but also the still more surprizing operations of nature, in perfecting a cure. She had employed four years, which, we see, were necessary to approximate the soft and solid parts of the coats of a great mass of purulent matter. Art had not tormented him, under the pretext of hastening a cure; and, if accidents have restrained the progress of Nature, she has been of herself able to surmount them, and they have been proportioned to the primary disorder. Nature acted in silence; our steps are quicker, but we often stumble. These truths add a new interest to the observations of the Baron.

We perceive in it the slow progress of nature, aided by the genius of art, when her own exertions were insufficient. We were aware by what gradual efforts nature cicatrizes, and and thus approximates parts the most distant, and repairs the greatest losses of hard and soft substances, even of the bones under many circumstances; but I am not aware that any one has observed and described, like the Baron Larrey, the general tendency of all the constituent parts of the breast, and the viscera which it contains, or which border upon it, to fill up the void left by the operation of the empyema, and the abundant extravasation of a fluid, and its residence in one of the pectoral cavities.

The Baron Larrey is not a person to dash forward in an unknown operation, for the sake of experiment: he is acquainted with all the resources of his art; and, if surgery is for him, as for another, a transcendent mean, he nevertheless owes his greatest success to the methodical use of a regimen varying, according to circumstances, to a just and sage modification of moral and physical objects which may have an influence on the patients, and the skilful administration of internal remedies, as well as to external applications; and his upright conduct proves that surgery is only useful when properly seconded by internal medicine—that their means ought to be inseparable, notwithstanding the prejudices of ignorance and intrigue, which, in our days, would establish a contrary system.

We decline repeating the medical treatment of Baron Larrey, in all the cases which have come under his care, in order that the length of our Report may not exceed that of the Notice.

We conclude by bestowing great approbation on the labour of the Baron Larrey; and, though the memoir is properly a continuation of what he has already printed, yet here is a special object, touching foreign bodies lodged in the breast; and it offers, besides, a new and interesting picture of the efforts of nature; we therefore propose to the Academy to insert it in the next volume of the Memoirs of learned Foreigners (des Memoirs des savans Etrangers).

Signed Deschamps.—Pelletan, Reporter.

The Academy approves the Report, and adopts the conclusions.

Certified to be conformably to the original.
The Perpetual Secretary, Counsellor of State, Chevalier of the Legion of Honour,
G. Cuvier.

